# Prevalence of schizophrenia spectrum disorders among adults in the Lazio region, Italy: use of an algorithm based on health information systems

**DOI:** 10.1192/j.eurpsy.2022.545

**Published:** 2022-09-01

**Authors:** A. Forastiere, S. Cascini, E. Calandrini, N. Agabiti, M. Davoli, A. Bargagli

**Affiliations:** Lazio Region, Department Of Epidemiology Of The Regional Health Service, Rome, Italy

**Keywords:** Schizophrenia spectrum disorders, Health Information System, Epidemiology, Prevalence

## Abstract

**Introduction:**

Mental healthcare provision is undergoing substantial reconfiguration in many regions of the world. Such changes require a broad evidence-based approach incorporating epidemiological data and information of local needs.

**Objectives:**

To estimate the prevalence of schizophrenia spectrum disorders (SSDs) in the Lazio region and its geographical distribution using the regional health information systems (HIS).

**Methods:**

Cases of SSDs (15-64-year-old) were identified using an algorithm based on data from the hospital discharge registry [ICD IX CM: 295, 297, 298 (excl. 298.0)] and the ticket exemption database [code 044], between 2006 and 2019. Crude, and age- and gender-specific prevalence estimates on December 31, 2019, were calculated. To compare prevalence between different areas within the region, we calculated age- and gender-adjusted prevalence rates

**Results:**

A total of 18,371 cases were identified. Crude prevalence rate was 4.29/1,000 (95% CI 4.29-4.30) and 5.93/1,000 (95% CI 5.92-5.949 for women and men, respectively. An increase in the prevalence rate by age was observed in both genders. The age- and gender-adjusted prevalence rate was 5.03/1,000 (95 % CI 4.96-5.10), with significant differences within the region, ranging from 4.25/1,000 in the province of Viterbo to 5.42/1,000 in the city of Rome and 6.02/1000 in the province of Frosinone.

**Conclusions:**

Our results showed that the overall prevalence of SSDs among adults in the Lazio region is similar to estimates published in prior reviews, but an uneven regional geographical distribution was observed. While possible underestimation must be considered, HIS represents a valuable source of information useful for epidemiological surveillance and healthcare planning.

**Disclosure:**

No significant relationships.

